# Four at a Time

**Published:** 1890-02

**Authors:** 


It is reported from Ingersoll, ten miles west of Texarkana, Ark., that a woman gave birth to four finely formed and well developed girl babies on Jan. 11. The mother is doing well, but the father is reported to be prostrated from sheer joy.

